# Implantation of Teeth

**Published:** 1887-01

**Authors:** G. L. Curtiss


### IMPLANTATION OF TEETH.

BY G. L. CURTISS, D. D. S.

Read before a Union Meeting of the 5th, 6th, 7th and 8th District Dental Societies of the State of New York, held at Rochester, October 26th and 27th, 1886.

With your permission I wish to very briefly bring before this society, for consideration and discussion, the subject of implantation, or the ingrafting of natural teeth into artificial sockets.

Dr. Wm. J. Younger, of San Francisco, is credited with being the first successful, if not the only present, operator in what has been called the only new thing of to-day in dental surgery, and the surprising and phenomenal success of that gentleman's experiments, I believe, makes this subject deserving of notice from this meeting.

The difficulties, with which you are all familiar, heretofore experienced by the dental profession in what is known as replantation and transplantation, seem to have been entirely overcome in what is now called by Dr. Younger implantation. The distinction between them is that the former operations consist in either putting back a tooth into a socket from which it has just been drawn, or the placing of a strange tooth in a socket from which one has been freshly extracted; while the latter operation, implantation, consists in making in the bone or process of the jaw an artificial socket, and ingrafting into such newly-made socket a stranger tooth that may have been extracted not a few hours only, but even months or years before.

Right here is one of the chief characteristics of the operation, namely, the successfully bringing into use and life teeth long extracted, which our profession has heretofore deemed an impracticable, if not an impossible thing to do; and if it be true as claimed, and I believe it is, that the cementum of the tooth will retain life for months, or even for a term of years, enabling it when placed in the socket to be revived and unite with the surrounding tissues and assume the normal conditions of a healthy tooth, then, as Dr. Younger truly says, implantation will soon be as firmly established among us as any operation requiring skill in dental surgery.

A paper upon the subject by Dr. Younger, and read before the San Francisco State Dental Society a little more than a year ago, so attracted the attention of the Chair of Dental Surgery of the University of Pennsylvania, that the subject was at once brought before the students of that institution as a new and very important step in dental science. That article, those lectures, which it was my privilege to hear, and subsequent articles upon the subject by Dr. Younger, so interested me that I resolved to put myself in communication with the San Francisco gentleman, and learn more of the details and practicability of the work. This I did, and with the aid of that gentleman's kind suggestions as to mode of operating, instruments, etc., I, a few weeks ago, decided I had made a sufficient study of the matter to safely undertake the work.

At the date of this writing I have performed five operations of this kind, in which I have implanted six teeth, and if I can intelligently judge from present indications, my work has been crowned with success as complete as ever experienced by Dr. Younger.

Three of the patients declare there was not even a soreness of the gums after the morning following the operation; all united in saying that they have observed no unusual or unpleasant results from the operation, and in every instance the teeth and gums present a healthy and natural appearance, the teeth fast becoming firm and solid as others in the mouth.

In my first case a slight swelling of the face appeared, which was soon removed, and this slight disturbance is all I have to report in the line of adverse results attending my operations.

My first was the placing of a superior second bicuspid in the place of a molar which had been extracted six years before. In my second case, the superior left bicuspid and the first and second molars had been lost seven years previous to the operation. I implanted a second bicuspid, and two weeks later two molars, completely filling this space with natural teeth.

My next patient had lost the superior lateral incisor. Being unable to obtain such a tooth, I took the root of an inferior bicuspid and attached a Richmond crown, filling the space and matching color, etc., to correspond with the rest of the teeth. My other patient had lost a lateral incisor from the effects of an abscess. There was a great deal of wasting of the alveoli, although the tooth had been out about two weeks only. In this case I took the root of an eye-tooth and attached a lateral incisor crown. Absorption had been so great that there seemed but little besides the gum remaining. I drilled into the old socket, finding only small traces of bone in which to imbed the new tooth. The tooth did not, under these circumstances, have the firmness of the others I have referred to at the time of the operation, but is now becoming quite firm, looks perfectly healthy, and all soreness is gone.

My mode of operating, briefly stated, is as follows: The tooth receives the ordinary treatment, as in transplantation, the pulp being removed and the pulp canal filled, and is placed in a solution of bichloride of mercury, which, in turn, is placed in warm water and raised to a temperature of 110° or 115° F., and allowed to remain from thirty minutes to an hour, according to the dryness of the cementum.

The gums I dissect away, laying bare the process, and with a spear-pointed drill start the socket on the line in which the tooth is to be placed, following this with larger drills and trepans, until the socket is the size of the tooth I wish to insert, when with corundum points I make smooth the walls of the socket. After syringing and thoroughly cleansing and disinfecting with a solution of bi-chloride of mercury, I insert the tooth, which should fit nicely and firmly in the new socket. I place closely fitting bands of gold or platinum about the implanted tooth and its immediate neighbors, thus forming a yoke to prevent any motion in or accident to the new tooth from mastication or otherwise. I have found two weeks a sufficiently long time, in most of the cases, to keep this on the teeth, the implanted one in that time becoming apparently almost as firm as the other teeth around it. I direct the patient to use a disinfecting mouth wash for a day or two after the operation.

Of course an operation so similar to those operations known as transplantation and replantation, in which so many failures have been experienced in the past, will not at once be received by the profession without some doubt as to its ultimate success, but if you will consider that in both those operations you often have to deal with diseased gums and sockets, in which it has been found impossible to keep alive the patient's own and natural teeth, and contrast the difficulties in making a foreign and stranger tooth attach and unite with such diseased gum with the more propitious circumstances of this operation, in which you seek to make a healthy tooth unite and grow in a perfectly healthy process, you will see the more favorable chance of success in the latter operation.

The greatest obstacle the writer deems likely to be met in these operations is the difficulty incident to getting suitable teeth from patients in whom the dentist has positive knowledge that there exists a high standard of health, with absence of impurities of the blood, etc.

With the exception, perhaps, of persons subject to hemorrhage and erysipelas, or in a low state of vitality, I belive every individual who wishes may safely have such operations performed, with a reasonable assurance of success.

